# Quantum–Classical Hybrid Systems and Ehrenfest’s Theorem

**DOI:** 10.3390/e25040602

**Published:** 2023-04-01

**Authors:** Alessandro Sergi, Daniele Lamberto, Agostino Migliore, Antonino Messina

**Affiliations:** 1 Dipartimento di Scienze Matematiche e Informatiche, Scienze Fisiche e Scienze della Terra, Università degli Studi di Messina, viale F. Stagno d’Alcontres 31, 98166 Messina, Italy; 2 Institute of Systems Science, Durban University of Technology, P.O. Box 1334, Durban 4000, South Africa; 3 Department of Chemical Sciences, University of Padova, Via Marzolo 1, 35131 Padova, Italy; 4 Dipartimento di Matematica ed Informatica, Università degli Studi di Palermo, Via Archirafi 34, 90123 Palermo, Italy

**Keywords:** quantum–classical dynamics, open quantum systems, quantum mechanics in phase space, Ehrenfest’s theorem, 03.65.-w, 05.30.-d, 03.65.Ca, 03.65.Sq, 11.15.Kc, 81Q20, 81S30, 82C10

## Abstract

The conceptual analysis of quantum mechanics brings to light that a theory inherently consistent with observations should be able to describe both quantum and classical systems, i.e., quantum–classical hybrids. For example, the orthodox interpretation of measurements requires the transient creation of quantum–classical hybrids. Despite its limitations in defining the classical limit, Ehrenfest’s theorem makes the simplest contact between quantum and classical mechanics. Here, we generalized the Ehrenfest theorem to bipartite quantum systems. To study quantum–classical hybrids, we employed a formalism based on operator-valued Wigner functions and quantum–classical brackets. We used this approach to derive the form of the Ehrenfest theorem for quantum–classical hybrids. We found that the time variation of the average energy of each component of the bipartite system is equal to the average of the symmetrized quantum dissipated power in both the quantum and the quantum–classical case. We expect that these theoretical results will be useful both to analyze quantum–classical hybrids and to develop self-consistent numerical algorithms for Ehrenfest-type simulations.

## 1. Introduction

The logical analysis of quantum mechanical phenomena highlights the general coexistence of classical and quantum systems and the consequent need for consistently describing them. The object of such a theory would be a quantum–classical hybrid (QCH), i.e., a system comprising a quantum and a classical interacting subsystem. QCHs are a subset of open quantum systems [1,2,3] and can also be called open quantum–classical systems [4].

Although not able to fully characterize the emergence of the classical regime [5,6,7], Ehrenfest’s theorem establishes the simplest formal link between the quantum and classical worlds. In fact, the quantum average over the positions and momenta of a quantum system in a time-dependent state performed using Ehrenfest’s theorem naturally leads to the emergence of QCHs described by classical-like variables together with Hamiltonian-like equations of motion [5,6,7]. Moreover, this theorem has been extended to relativistic quantum theory [8].

Here, we developed Ehrenfest’s theorem for quantum bipartite systems, as the latter play a special role in the generation of QCHs. We specialized our theorem to the case of a quantum bipartite system comprising light and heavy particles. Such systems can be studied in terms of an operator-valued Wigner function [9,10,11,12,13,14] and its equation of motion defined by means of a quantum–classical bracket [15,16,17,18,19,20,21]. One can also define QCHs by means of the partial Wigner transform of the density matrix with respect to the heavy coordinates [22]. In this way, one obtains an operator-valued Wigner function. The equation of motion is obtained by applying the same partial transform to the quantum Liouville equation and linearizing the result. The law of evolution is then defined by an antisymmetric quantum–classical bracket [15,16,17,18,19,20,21]. Using the operator-valued Wigner function [9,10,11,12,13,14] and the quantum–classical bracket [15,16,17,18,19,20,21], we derived Ehrenfest’s theorem for QCHs. Notably, both for the quantum bipartite system and for the QCH, we found that the time variation of the quantum/quantum–classical ensemble average of the energy (i.e., the average energy dissipation) is equal to the average of the symmetrized power.

From a practical perspective, QCH are also important, as already noted, to approximate quantum many-body problems involving systems with mixtures of particles with light and heavy masses. There are various theoretical formalisms for describing QCHs, including approaches based on the path integral formalism [23,24], the linear QCH dynamics [25,26], hybrid quantum–classical master equations [27], and quantum–classical ensembles in configuration space [28,29]. Using the results of [30,31], it was suggested in [32,33] that one could devise a representation of QCHs where both quantum and classical variables are defined in phase space. This is achieved using conditional tomographic probability distributions [34]. We adopted here a formalism for QCH dynamics based on an operator-valued Wigner function that depends parametrically on the phase space coordinates of the classical system [9,10,11,12,13,14]. In passing, we note that there are also approaches for studying the semi-classical limit of the Wigner function, (see, e.g., [35]). However, here, in line with the approach of [9,10,11,12,13,14,15,16,17,18,19,20,21], we considered QCHs to exist in their own right, regardless of any limiting procedure. The motivations for this perspective will be discussed in the following.

The paper is organized as follows. In Section 2, the role of QCHs in quantum mechanics is discussed with emphasis on both known and lesser known issues. The standard Ehrenfest’s theorem for an isolated system is sketched in Appendix A. In Section 3, we derive the form of Ehrenfest’s theorem for a quantum bipartite system. In this case, both quantum subsystems are open with respect to each other. Thus, while the average energy of the total system is a constant of motion, the energy of each separate subsystem is not conserved. We show that the change in time of the average energy is equal to the average of the anticommutator of the quantum dissipated energy. In Section 4, we consider the dynamics of QCHs. These are a particular type of quantum open systems where one of the two systems is classical. There are many situations in which QCHs appear, and we also discuss them in Section 2. The equation of motion of QCHs is introduced in Section 4, where its algebraic properties are also discussed. Section 5 is devoted to the derivation of Ehrenfest’s theorem for QCHs.

## 2. Relation between Quantum and Classical Worlds

After almost one century from the birth of quantum mechanics (QM) and the inception of the orthodox interpretation (OI) [36,37], a large part of the scientific community is still searching for a more satisfactory understanding of the theory’s ontology [38,39]. From a mathematical point of view, QM is an elegant theory. The work of Dirac enlightens QM’s structural algebraic connection with classical mechanics, while QM’s probabilistic rules, together with the principle of state reduction, provide solid machinery for the theoretical predictions of the behavior of physical systems. However, the situation is not so bright from the conceptual point of view. Landau [40] noted that QM needs classical mechanics in order to be formulated. The quantum formalism is obtained by first considering a classical system and, afterwards, quantizing it: except in a few cases, nobody has so far managed to directly write quantum equations of motion without taking the “classical step” first. To capture this state of affairs, Landau wrote that “quantum mechanics is not logically closed” [40].

The role of classical systems is not only to be some kind of algebraic blueprint for the formulation of QM. Ultimately, a theory must predict the results of measurements. In the present understanding of QM, the properties of a quantum system can only be measured by classical instruments. Accordingly, quantum systems and classical systems, i.e., instruments, must coexist in the study of quantum systems. QM describes the action of classical instruments by means of a stochastic evolution, i.e., the collapse of the state [36,37,40,41,42,43], which is a non-linear process. In fact, it is well known that, during the measurement, linear coherent equations of motion are no longer valid. This is the famous “measurement problem” [36,37,40,41,42,43]. We can see that a quantum–classical hybrid (QCH) naturally arises from a quantum subsystem interacting with an external classical instrument. Moreover, the quantum system’s environment can also act as a measuring apparatus, thus manifesting classical properties.

**Figure 1:**
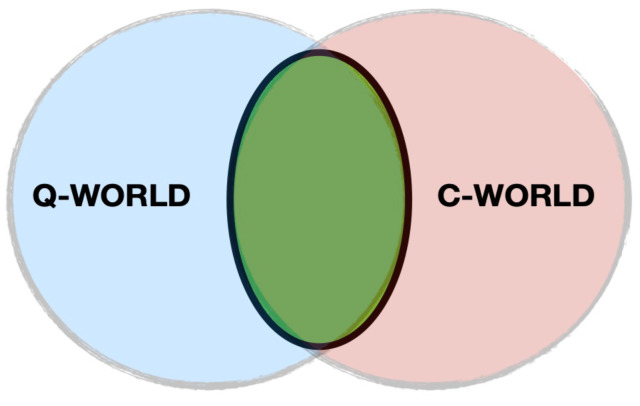
Relation between the quantum and the classical worlds. The classical world is not contained in a larger and more fundamental quantum world. Instead, there is only an overlap between the two worlds. In the picture, the cyan quantum world (Q-world) overlaps with the light orange classical world (C-world), and the green area represents their intersection.

Some interpretations of quantum mechanics deal with the measurement problem in a somewhat more consistent way, e.g., the de Broglie-Bohm approach [44], Cramer’s, time-symmetric, transactional interpretations of QM [45,46], and its possibilist variant [47]. In doing so, these theories pay the price of making the non-local character of QM even more apparent. As is well known, Bohm’s potential is non-local in configuration space [44]. The translational interpretation of QM [45,46,47] is based on an even more astounding non-locality, according to which interactions are highly non-causal because the action of the future on the past carries the same weight as the action of the past on the future. Moreover, they still suffer from the fact that they are logically open, i.e., the equations are exactly the same as the standard QM, and they cannot be written without imagining a model of the classical world first. Moreover, they do not really solve the problem of rigorously explaining how classical phenomena emerge from quantum ones. The possibility of the existence of classical variables representing a measurement apparatus is linked to the existence of classical states (i.e., non-linear chaotic states) that cannot be quantized and, conversely, quantum states (i.e., stationary states) that do not admit a classical limit [44]. This situation is depicted in Figure 1: The classical world is not contained in a larger and more fundamental quantum world. Instead, there is only an overlap between the two worlds [44]. Nevertheless, the “classical” system is composed of quantum particles, and the theory does not tell us when a system of quantum particles becomes a classical object. This fact supports the reality of QCHs.

Besides the measurement problem, another argument in favor of the fundamental role of QCHs in physics is found within certain approaches to quantum gravity. QCHs are necessary for describing quantum processes taking place on classical curved manifolds [48,49,50,51]. Due to the present lack of a definitive theory of quantum gravity [52,53,54,55,56,57], one must resort to a quantum–classical theory of motion [50,51]. QCHs have a true foundational status for theories that look at quantum gravity as an emergent phenomenon [58,59,60,61,62,63]. In particular, Roger Penrose proposed an approach for the quantum-to-classical limit, called objective reduction (OR), which involves an unquantized gravity field coupled to quantum matter [64,65,66]. OR explains the quantum-to-classical limit by turning the idea of quantizing gravity on its head: indeed, Penrose proposed to *gravitize* quantum theory [64,65,66]. Basically, the idea behind OR is that all quantum particles are locally coupled to their own gravitational field. Because of this coupling, an unstable entangled state of particles is created together with particles’ gravitational self-field. Essentially, different gravitational metrics imply different kinds of universes. In OR, it is assumed that this is not possible and that the more non-local the separate particles’ states become, the more unstable is their entangled state with the metric self-field. In a certain sense, the self-field is intrinsically classical and measures the quantum superposition of states that generates it. Despite the superficial similarity with the OI, OR assigns the collapse of the wave function to the internal dynamics of the system (coupled to the local gravitational self-field), rather than resorting to absolutely unpredictable interactions with classical objects that are external to the system. Such a mechanics necessitates QCHs.

When a quantum many-body system comprises light and heavy particles, approximation as a QCH can enable efficient numerical calculations [67,68,69,70,71,72,73,74,75]. Our theoretical approach uses an operator-valued Wigner function [9,10,11,12,13,14], thus enabling a suitable representation of mixed states and making the theory amenable to the derivation of controllable approximations of numerical algorithms [75,76]. Approximations of many-body problems in terms of QCHs most naturally appear in the description of chemical and biochemical reactions in gases, fluids, and gels. One example is given by superoxide dismutase (SOD) [77,78,79,80]. SOD is an enzyme whose action favors the reduction of O${}_{2}^{-}$ into O${}_{2}$ and H${}_{2}$O${}_{2}$. In Cu, Zn SODs, the whole physical process can be approximately analyzed in terms of a classical diffusive motion of the superoxide to the quantum reactive center, i.e., the Cu atom, inside the SOD protein, which is able to transfer quantum electrons between the different atoms involved in the reactions.

## 3. Ehrenfest Theorem for Bipartite Systems

Before tackling QCHs, we take into consideration bipartite quantum systems. In the next section, we will see that a QCH can be obtained via the classical approximation of one of the two components of the otherwise completely quantum bipartite system. Thus, we now consider a bipartite system SB, composed of subsystems S and B. The position and momentum operators of subsystem S are denoted by $\hat{r}$ and $\hat{p}$, respectively. They are also denoted collectively as $\hat{x}=\left(\hat{r},\hat{p}\right)$. As for subsystem B, its position and momentum operators are written as $\hat{R}$ and $\hat{P}$, respectively. Similarly, we use the notation $\hat{X}=\left(\hat{R},\hat{P}\right)$ for subsystem B. Using a compact notation, the position and momentum operators of SB are globally written as $\hat{\chi }=\left(\hat{x},\hat{X}\right)$. Note that we are using a multidimensional notation akin to that introduced in [22]. Accordingly, $\left(\hat{r},\hat{p}\right)\equiv \left({\hat{r}}_{1},\ldots ,{\hat{r}}_{n},{\hat{p}}_{1},\ldots ,{\hat{p}}_{n}\right)$ and $\left(\hat{R},\hat{P}\right)\equiv \left({\hat{R}}_{1},\ldots ,{\hat{R}}_{N},{\hat{P}}_{1},\ldots ,{\hat{P}}_{N}\right)$, where *n* is the number of configurational coordinates of system S and *N* is the number of configurational coordinates of system B, respectively. In this way, $\hat{x}$ is the multidimensional notation for ${\hat{x}}_{j}$, with j=1,…,2n, and $\hat{X}$ stands for ${\hat{X}}_{K}$, K=1,…,N. Thus, the multidimensional symbol $\hat{\chi }$ is the compact notation for ${\hat{\chi }}_{\alpha }$, with α=1,…,2(n+N), describing all the canonical operators of the bipartite system. This means that our theory is naturally valid for multidimensional systems.

We assumed that the Hamiltonian operator of the coupled system can be written as

(1) $${\hat{H}}_{\mathrm{SB}}\left(\hat{\chi }\right)={\hat{H}}_{S}\left(\hat{x}\right)+{\hat{H}}_{B}\left(\hat{X}\right)+{\hat{V}}_{\mathrm{SB}}\left(\hat{r},\hat{R}\right)\;,$$

where the Hamiltonian operators of subsystems S and B are

(2) $${\hat{H}}_{S}\left(\hat{x}\right)=\frac{{\hat{p}}^{2}}{2m}+{\hat{V}}_{S}\left(\hat{r}\right)\;,\;\;\;{\hat{H}}_{B}\left(\hat{X}\right)=\frac{{\hat{P}}^{2}}{2M}+{\hat{V}}_{B}\left(\hat{R}\right)\;,$$

respectively, and ${\hat{V}}_{\mathrm{SB}}\left(\hat{r},\hat{R}\right)$ is the coupling potential.

Ehrenfest’s theorem for the composite system is obtained by using the same kind of reasoning adopted in the case of the isolated system S, which is treated in Appendix A. A general quantum state of system SB is represented by the density matrix ${\hat{f}}_{\mathrm{SB}}\left(t\right)$ [81,82,83]. In the Schrödinger scheme of motion, ${\hat{f}}_{\mathrm{SB}}\left(t\right)$ obeys the equation of motion:

(3) $$\begin{array}{ccc} \frac{d{\hat{f}}_{\mathrm{SB}}\left(\hat{\chi },t\right)}{dt} & = & -\frac{i}{\hbar }\left[{\hat{H}}_{\mathrm{SB}}\left(\hat{\chi }\right),{\hat{f}}_{\mathrm{SB}}\left(\hat{\chi },t\right)\right]\;. \end{array}$$

The time evolution equations of ${\langle r\rangle }_{t}$ and ${\langle R\rangle }_{t}$ are given by

(4) $$\begin{array}{ccc} \frac{d}{dt}{\langle \hat{r}\rangle }_{t} & = & {\mathrm{Tr}}_{\mathrm{SB}}\left(\left[{\hat{f}}_{\mathrm{SB}}\left(\hat{\chi },t\right),\hat{r}\right]\right)=\frac{{\langle \hat{p}\rangle }_{t}}{m}\;, \end{array}$$

(5) $$\begin{array}{ccc} \frac{d}{dt}{\langle \hat{R}\rangle }_{t} & = & \frac{{\langle \hat{P}\rangle }_{t}}{m}\;. \end{array}$$

where ${\mathrm{Tr}}_{\mathrm{SB}}$ represents the trace over both subsystems S and B. For the expectation values of momenta, one finds

(6) $$\begin{array}{ccc} \frac{d{\langle \hat{p}\rangle }_{t}}{dt} & = & -{\langle \frac{\partial {\hat{V}}_{S}\left(r\right)}{\partial r}{|}_{r\to \hat{r}}\rangle }_{t}-{\langle \frac{\partial {\hat{V}}_{\mathrm{SB}}\left(r,\hat{R}\right)}{\partial r}{|}_{r\to \hat{r}}\rangle }_{t}\;, \end{array}$$

(7) $$\begin{array}{ccc} \frac{d{\langle \hat{P}\rangle }_{t}}{dt} & = & -{\langle \frac{\partial {\hat{V}}_{B}\left(R\right)}{\partial R}{|}_{R\to \hat{R}}\rangle }_{t}-{\langle \frac{\partial {\hat{V}}_{\mathrm{SB}}\left(\hat{r},R\right)}{\partial R}{|}_{R\to \hat{R}}\rangle }_{t}\;. \end{array}$$

Equations (4)–(7) establish Ehrenfest’s theorem in the case of a bipartite quantum system. Equations (4) and (5) are identical to those one would obtain in the case of ${\hat{V}}_{\mathrm{SB}}\left(\hat{r},R\right)=0$. Instead, Equations (6) and (7) contain the mutual backreaction of a system upon the other. This backreaction is given by the averages of the quantum forces $-{\langle \partial_{r}{\hat{V}}_{\mathrm{SB}}\left(r,\hat{R}\right){|}_{r\to \hat{r}}\rangle }_{t}$ and $-{\langle \partial_{R}{\hat{V}}_{\mathrm{SB}}\left(r,\hat{R}\right){|}_{R\to \hat{R}}\rangle }_{t}$, where $\partial_{r}=\partial /\partial r$ and $\partial_{R}=\partial /\partial R$.

Because of the coupling ${\hat{V}}_{\mathrm{SB}}\left(\hat{r},\hat{R}\right)$, the average value of ${\hat{H}}_{S}$ is not conserved. The variation of the average value ${\langle {\hat{H}}_{S}\rangle }_{t}$ over time is

(8) $$\begin{array}{ccc} \frac{d}{dt}{\langle {\hat{H}}_{S}\left(\hat{x}\right)\rangle }_{t} & = & {\mathrm{Tr}}_{\mathrm{SB}}(\left(\frac{d}{dt}{\hat{f}}_{\mathrm{SB}}\left(\hat{\chi },t\right)\right){\hat{H}}_{S}\left(\hat{x}\right))\\ & = & -\frac{i}{\hbar }{\mathrm{Tr}}_{\mathrm{SB}}\left(\left[{\hat{H}}_{\mathrm{SB}}\left(\hat{x}\right),{\hat{f}}_{\mathrm{SB}}\left(\hat{\chi },t\right)\right]{\hat{H}}_{S}\left(\hat{x}\right)\right)\\ & = & -\frac{i}{\hbar }{\mathrm{Tr}}_{\mathrm{SB}}\left({\hat{f}}_{\mathrm{SB}}\left(\hat{\chi },t\right)\left[{\hat{H}}_{S}\left(\hat{x}\right),{\hat{H}}_{\mathrm{SB}}\left(\hat{\chi }\right)\right]\right)\\ & = & -\frac{i}{\hbar }{\mathrm{Tr}}_{\mathrm{SB}}\left({\hat{f}}_{\mathrm{SB}}\left(\hat{\chi },t\right)\left[\frac{{\hat{p}}^{2}}{2m},{\hat{V}}_{\mathrm{SB}}\left(\hat{r},\hat{R}\right)\right]\right)\\ & = & -{\mathrm{Tr}}_{\mathrm{SB}}\left({\hat{f}}_{\mathrm{SB}}\left(\hat{\chi },t\right)\left[\frac{\hat{p}}{2m}\cdot \frac{\partial {\hat{V}}_{\mathrm{SB}}\left(r,\hat{R}\right)}{\partial r}{{|}_{r\to \hat{r}}+\frac{\partial {\hat{V}}_{\mathrm{SB}}\left(r,\hat{R}\right)}{\partial r}|}_{r\to \hat{r}}\;\cdot \frac{\hat{p}}{2m}\right]\right)\\ & = & -\frac{1}{2}{\langle \left\{\frac{\partial {\hat{V}}_{\mathrm{SB}}\left(r,\hat{R}\right)}{\partial r}{|}_{r\to \hat{r}}\;,\frac{\hat{p}}{m}\right\}\rangle }_{t}\;, \end{array}$$

where · is the multidimensional scalar product and {…,…} is the anticommutator of the quantum force acting on system S because of its coupling to system B. The term $-\partial_{r}\hat{V}\left(r,\hat{R}\right){|}_{r\to \hat{r}}\;\cdot \hat{p}/m$ represents the quantum dissipated power by system S. The anticommutator in the rhs of Equation (8) obeys Weyl’s ordering rule. We believe that the derived Equation (8) is an elegant formula providing a structure as close as possible to the classical formalism, while taking into account quantum algebra in a way that can readily be expressed in terms of Wigner–Weyl’s formalism [84,85,86,87,88,89]. Since the Hamiltonian ${\hat{H}}_{\mathrm{SB}}\left(\hat{\chi }\right)$ is symmetric under the transformation $\hat{x}⇄\hat{X}$, Equations (4)–(8) take the same form for subsystem B.

In the next section, we show how the previous analysis can be adapted to QCHs. We will find that the formulas retain their structure after a suitable reinterpretation of the symbols involved.

## 4. The Dynamics of Quantum–Classical Hybrids

As discussed in the Introduction, there is a variety of situations in which it is necessary to adopt a hybrid quantum–classical description of a bipartite system. When M≫m, it follows that Λ≪λ, where Λ and λ are the de Broglie wavelengths of B and S, respectively. Thus, one can disregard quantum effects on the dynamics of B and take the classical approximation $\hat{R}\to R$ and $\hat{P}\to P$. This means that the Hamiltonian of B in Equation (2) becomes purely classical:

(9) $$H_{B}\left(X\right)=\frac{P^{2}}{2M}+V_{B}\left(R\right)\;,$$

while the coupling potential becomes a hybrid operator ${\tilde{V}}_{\mathrm{SB}}\left(\hat{r},R\right)$. The Hamiltonian operator of S is still defined by Equation (2). Hence, the total Hamiltonian of SB:

(10) $${\tilde{H}}_{\mathrm{SB}}\left(\tilde{\chi }\right)={\hat{H}}_{S}\left(\hat{x}\right)+H_{B}\left(X\right)+{\tilde{V}}_{\mathrm{SB}}\left(\hat{r},R\right)$$

defines a QCH, i.e., a hybrid system where classical and quantum “variables” are mixed. In Equation (10), the tilde on the symbols $\tilde{\chi }=\left(\hat{x},X\right)$ and ${\tilde{H}}_{\mathrm{SB}}\left(\tilde{\chi }\right)$ stands for their double dependence on both quantum operators and classical parameters. Since the classical parameters *X* correspond to the (R,P) positions and momenta of B, they can be identified with the phase space coordinates of B. Since P≪p (because M≫m), one can take an Eulerian point of view, according to which the quantum motion of S occurs at high frequencies, while B, from the point of view of S, appears frozen. So far, we have considered QCHs following the historical development and via physical intuition. However, some years ago, it was proven that QCHs can be obtained through a specific first-order approximation of the partially Wigner-transformed commutator of a quantum bipartite system. This is shown in Appendix B.

In the Schrödinger scheme of motion (while hybrid or quantum operators are constant over time), the state operator of the QCH system, ${\tilde{f}}_{\mathrm{SB}}\left(\tilde{\chi },t\right)$, obeys an equation for its time evolution that couples the quantum degrees of freedom to the classical parameters *X*:

(11) $$\begin{array}{ccc} \frac{\partial }{\partial t}{\tilde{f}}_{\mathrm{SB}}\left(\tilde{\chi },t\right) & = & -\frac{i}{\hbar }[{\tilde{H}}_{\mathrm{SB}}\left(\tilde{\chi }\right),{\tilde{f}}_{\mathrm{SB}}\left(\tilde{\chi },t\right)]+\frac{1}{2}\sum_{kl}J_{kl}(\nabla_{k}{\tilde{H}}_{\mathrm{SB}}\left(\tilde{\chi }\right))\nabla_{l}{\tilde{f}}_{\mathrm{SB}}\left(\tilde{\chi },t\right)\\ & & -\frac{1}{2}\sum_{mn}J_{mn}(\nabla_{m}{\tilde{f}}_{\mathrm{SB}}\left(\tilde{\chi },t\right))\nabla_{n}{\tilde{H}}_{\mathrm{SB}}\left(\tilde{\chi }\right)\\ & \equiv & -\frac{i}{\hbar }\tilde{[}{\tilde{H}}_{\mathrm{SB}}\left(\tilde{\chi }\right),{\tilde{f}}_{\mathrm{SB}}\left(\tilde{\chi },t\right)\tilde{]}\;. \end{array}$$

The symbol $\nabla =(\partial_{R},\partial_{P})$ represents the phase space gradient, and J is the symplectic matrix [90]:

(12) $$J=\left[\begin{array}{cc} 0 & 1\\ -1 & 0 \end{array}\right]\;.$$

The first term in the rhs of Equation (11) is the commutator between ${\tilde{H}}_{\mathrm{SB}}\left(\tilde{\chi }\right)$ and ${\tilde{f}}_{\mathrm{SB}}\left(\tilde{\chi },t\right)$. The commutator can also be written in terms of the symplectic matrix [19,20,21] J:

(13) $$\left[\begin{array}{cc} {\tilde{H}}_{\mathrm{SB}}\left(\tilde{\chi }\right) & {\tilde{f}}_{\mathrm{SB}}\left(\tilde{\chi },t\right) \end{array}\right]J\left[\begin{array}{c} {\tilde{H}}_{\mathrm{SB}}\left(\tilde{\chi }\right)\\ {\tilde{f}}_{\mathrm{SB}}\left(\tilde{\chi },t\right) \end{array}\right]\;.$$

It is easy to verify that the last two terms of Equation (11) are the Poisson brackets of ${\tilde{H}}_{\mathrm{SB}}\left(\hat{x},X\right)$ and ${\tilde{f}}_{\mathrm{SB}}\left(\tilde{\chi },t\right)$ defined so that their combination is antisymmetric.

All three terms in the rhs of Equation (11) define the quantum–classical bracket $\tilde{[}\ldots ,\ldots \tilde{]}$ [15,16,17,18,19,20,21]. There are deep physical reasons for postulating that the dynamics of the state operator of QCHs is defined by the quantum–classical bracket [15,16,17,18,19,20,21]. Such a bracket is a quasi-Lie bracket because it obeys all the properties of Lie brackets, but the Jacobi relation:

(14) $$\begin{array}{c} \tilde{[}{\tilde{O}}_{1},\tilde{[}{\tilde{O}}_{2},{\tilde{O}}_{3}\tilde{]}\tilde{]}+\tilde{[}{\tilde{O}}_{3},\tilde{[}{\tilde{O}}_{1},{\tilde{O}}_{2}\tilde{]}\tilde{]}+\tilde{[}{\tilde{O}}_{2},\tilde{[}{\tilde{O}}_{3},{\tilde{O}}_{1}\tilde{]}\tilde{]}\neq 0\;. \end{array}$$

Equation (14) represents the failure of the Jacobi relation of Lie algebras (wherein it is an identity) in the quasi-Lie algebra of quantum–classical brackets. In general, this implies that quasi-Lie algebras do not obey time translation invariance. In the case of QCHs, the evolution in time will inevitably mix quantum and classical operators so that, even in the cases when S and B are initially uncorrelated, the quantum–classical bracket will inevitably produce a QCH (when, of course, ${\tilde{V}}_{\mathrm{SB}}\left(\hat{r},R\right)\neq 0$). We now illustrate this point explicitly. Let us assume that the state operator of the QCH is that of an uncorrelated initial state of S and B. This is written as

(15) $${\tilde{f}}_{\mathrm{SB}}\left(\tilde{\chi }\right)=f_{B}\left(X\right)\;{\hat{f}}_{S}\left(\hat{x}\right)\;.$$

Upon propagating the state of Equation (15) over time by

(16) $${\tilde{f}}_{\mathrm{SB}}\left(\tilde{\chi },t\right)=e^{-\left(i/\hbar \right)\tilde{[}{\tilde{H}}_{\mathrm{SB}}\left(\tilde{\chi }\right),\ldots \tilde{]}}{\tilde{f}}_{\mathrm{SB}}\left(\tilde{\chi }\right)\;,$$

the “sector” of the quantum variables $\hat{x}$ and the “sector” of the phase space parameters *X* become unavoidably mixed. Hence, even if the quasi-Lie hybrid bracket in Equation (11) conserves the energy and the probability (it can be easily verified that the propagator in Equation (16) is unitary), the quasi-Lie structure of the algebra obeyed by QCHs introduces a form of irreversibility: the “partition” of the QCH defines a particular type of open quantum system.

## 5. Ehrenfest’s Theorem for Quantum–Classical Hybrids

Average values of the variables of the QCH are calculated as

(17)〈〈O˜(χ˜)〉〉t=TrS∫ΩdXf˜SB(χ˜,t)O˜(χ˜)(18)=T˜rSBf˜SB(χ˜,t)O˜(χ˜)

The double brackets in the lhs of Equation (17) imply a double averaging, i.e., a partial trace over the quantum degrees of freedom and an integral over the phase space parameters of the QCH. In the rhs of Equation (17), ${\mathrm{Tr}}_{S}\left(\ldots \right)$ stands for the partial trace over $\hat{x}$, while $\int_{\Omega }dX\;\ldots$ is clearly the phase space integral. Both operations are indicated by $\tilde{T}r_{\mathrm{SB}}\left(\ldots \right)$ in the rhs of (18).

To derive Ehrenfest’s theorem, we consider

(19) $$\begin{array}{ccc} \frac{d}{dt}{\langle \langle \hat{r}\rangle \rangle }_{t} & = & \tilde{T}r_{\mathrm{SB}}\left(\frac{\partial }{\partial t}\tilde{f}\left(\tilde{\chi },t\right)\hat{r}\right)\\ & = & -\frac{i}{\hbar }\tilde{T}r_{\mathrm{SB}}\left(\left[{\tilde{H}}_{\mathrm{SB}}\left(\tilde{\chi }\right),\tilde{f}\left(\tilde{\chi },t\right)\right]\hat{r}\right)\\ & + & \frac{1}{2}\tilde{T}r_{\mathrm{SB}}\left[\sum_{jk}J_{jk}\left(\nabla_{j}{\tilde{H}}_{\mathrm{SB}}\left(\tilde{\chi }\right)\right)\left(\nabla_{k}\tilde{f}\left(\tilde{\chi },t\right)\right)\hat{r}\right]\\ & - & \frac{1}{2}Tr_{\mathrm{SB}}\left[\sum_{mn}J_{mn}\left(\nabla_{m}\tilde{f}\left(\tilde{\chi },t\right)\right)\left(\nabla_{n}{\tilde{H}}_{\mathrm{SB}}\left(\tilde{\chi }\right)\right)\hat{r}\right] \end{array}$$

We also calculate

(20) $$\begin{array}{ccc} -\frac{i}{\hbar }\tilde{T}r_{\mathrm{SB}}\left(\left[{\tilde{H}}_{\mathrm{SB}}\left(\tilde{\chi }\right),\tilde{f}\left(\tilde{\chi },t\right)\right]\hat{r}\right) & = & -\frac{i}{\hbar }\tilde{T}r_{\mathrm{SB}}\left[{\tilde{H}}_{\mathrm{SB}}\left(\tilde{\chi }\right)\tilde{f}\left(\tilde{\chi },t\right)\hat{r}-\tilde{f}\left(\tilde{\chi },t\right){\tilde{H}}_{\mathrm{SB}}\left(\tilde{\chi }\right)\hat{r}\right]\\ & = & -\frac{i}{\hbar }\tilde{T}r_{\mathrm{SB}}\left[\tilde{f}\left(\tilde{\chi },t\right)\hat{r}{\tilde{H}}_{\mathrm{SB}}\left(\tilde{\chi }\right)-\tilde{f}\left(\tilde{\chi },t\right){\tilde{H}}_{\mathrm{SB}}\left(\tilde{\chi }\right)\hat{r}\right]\\ & = & -\frac{i}{\hbar }\tilde{T}r_{\mathrm{SB}}\tilde{f}\left(\tilde{\chi },t\right)\left[\hat{r},{\tilde{H}}_{\mathrm{SB}}\left(\tilde{\chi }\right)\right]\\ & = & -\frac{i}{\hbar }\tilde{T}r_{\mathrm{SB}}\left(\tilde{f}\left(\tilde{\chi },t\right)\left[\hat{r},\frac{p^{2}}{2m}\right]\right)\\ & = & \tilde{T}r_{\mathrm{SB}}\left(\tilde{f}\left(\tilde{\chi },t\right)\frac{p}{m}\right)=\frac{{\langle \langle \hat{p}\left(t\right)\rangle \rangle }_{t}}{m}\;. \end{array}$$

The second term in the rhs of Equation (19) is

(21) $$\begin{array}{ccc} \tilde{T}r_{\mathrm{SB}}\left[\sum_{jk}J_{jk}\left(\nabla_{j}{\tilde{H}}_{\mathrm{SB}}\left(\tilde{\chi }\right)\right)\left(\nabla_{k}\tilde{f}\left(\tilde{\chi },t\right)\right)\hat{r}\right] & = & -\tilde{T}r_{\mathrm{SB}}\left[\sum_{j,k}J_{jk}\left(\nabla_{kj}^{2}{\tilde{H}}_{\mathrm{SB}}\left(\tilde{\chi }\right)\right)\tilde{f}\left(\tilde{\chi },t\right)\hat{r}\right]=0\;. \end{array}$$

Equation (21) follows from the fact that $\nabla_{k}\hat{r}=0$ and $\sum_{jk}J_{jk}\nabla_{jk}^{2}{\hat{H}}_{\mathrm{SB}}\left(\tilde{\chi }\right)=0$, because it is the trace of the product of an antisymmetric matrix by a symmetric one. Analogously, we find that

(22) $$\begin{array}{ccc} \tilde{T}r_{\mathrm{SB}}\left[\sum_{jk}J_{jk}(\nabla_{j}\tilde{f}\left(\tilde{\chi },t\right))(\nabla_{k}{\tilde{H}}_{\mathrm{SB}}\left(\tilde{\chi }\right))\hat{r}\right] & = & 0\;. \end{array}$$

To complete the derivation of Ehrenfest’s theorem in the case of a QCH, we must consider the time derivative of ${\langle \langle \hat{p}\rangle \rangle }_{t}$. Let us consider now

(23) $$\begin{array}{ccc} \frac{d}{dt}{\langle \langle \hat{p}\rangle \rangle }_{t} & = & \tilde{T}r_{\mathrm{SB}}\left(\frac{\partial }{\partial t}\tilde{f}\left(\tilde{\chi },t\right)\hat{p}\right)\\ & = & -\frac{i}{\hbar }\tilde{T}r_{\mathrm{SB}}([{\tilde{H}}_{\mathrm{SB}}\left(\tilde{\chi }\right),\tilde{f}\left(\tilde{\chi },t\right)]\hat{p})\\ & + & \frac{1}{2}\tilde{T}r_{\mathrm{SB}}[\sum_{jk}J_{jk}(\nabla_{j}{\tilde{H}}_{\mathrm{SB}}\left(\tilde{\chi }\right))(\nabla_{k}\tilde{f}\left(\tilde{\chi },t\right))\hat{p}]\\ & - & \frac{1}{2}\tilde{T}r_{\mathrm{SB}}[\sum_{mn}J_{mn}(\nabla_{m}\tilde{f}\left(\tilde{\chi },t\right))(\nabla_{n}{\tilde{H}}_{\mathrm{SB}}\left(\tilde{\chi }\right))\hat{p}]\;. \end{array}$$

We calculate the three terms in the rhs of Equation (23). For the the average of the commutator, we obtain

(24) $$\begin{array}{ccc} -\frac{i}{\hbar }\tilde{T}r_{SB}([{\tilde{H}}_{\mathrm{SB}}\left(\tilde{\chi }\right),\tilde{f}\left(\tilde{\chi },t\right)]\hat{p}) & = & -\frac{i}{\hbar }\tilde{T}r_{\mathrm{SB}}(\tilde{f}\left(\tilde{\chi },t\right)[\hat{p},{\tilde{H}}_{\mathrm{SB}}\left(\tilde{\chi }\right)])\\ & = & -\frac{i}{\hbar }{\langle \langle [\hat{p},{\hat{V}}_{S}\left(\hat{r}\right)]\rangle \rangle }_{t}-\frac{i}{\hbar }{\langle \langle [\hat{p},{\tilde{V}}_{\mathrm{SB}}\left(\hat{r},R\right)]\rangle \rangle }_{t}\; \end{array}$$

while for the other two terms on the rhs of Equation (23), we have

(25) $$\begin{array}{ccc} \tilde{T}r_{\mathrm{SB}}[\sum_{jk}J_{jk}(\nabla_{j}{\tilde{H}}_{\mathrm{SB}}\left(\tilde{\chi }\right))(\nabla_{k}\tilde{f}\left(\tilde{\chi },t\right))\hat{p}] & = & 0;, \end{array}$$

(26) $$\begin{array}{ccc} \tilde{T}r_{\mathrm{SB}}[\sum_{mn}J_{mn}(\nabla_{m}\tilde{f}\left(\tilde{\chi },t\right))(\nabla_{n}{\tilde{H}}_{\mathrm{SB}}\left(\tilde{\chi }\right))\hat{p}] & = & 0\;. \end{array}$$

Finally, collecting the formulas for $\left(d/dt\right){\langle \langle \hat{r}\rangle \rangle }_{t}$ and $\left(d/dt\right){\langle \langle \hat{p}\rangle \rangle }_{t}$, the Ehrenfest theorem in a classical bath is given by

(27) $$\begin{array}{ccc} \frac{d}{dt}{\langle \langle \hat{r}\rangle \rangle }_{t} & = & {\langle \langle \frac{\hat{p}\left(t\right)}{m}\rangle \rangle }_{t}\;, \end{array}$$

(28) $$\begin{array}{ccc} \frac{d}{dt}{\langle \langle \hat{p}\rangle \rangle }_{t} & = & -{\langle \langle \frac{d{\hat{V}}_{S}\left(r\right)}{dr}\rangle \rangle }_{t}-{\langle \langle \frac{\partial {\tilde{V}}_{\mathrm{SB}}\left(\hat{r},R\right)}{\partial R}\rangle \rangle }_{t}\;. \end{array}$$

To complete the derivation of Ehrenfest’s theorem for the QCH, we also have to calculate

(29) $$\begin{array}{ccc} \frac{d}{dt}{\langle \langle R\rangle \rangle }_{t} & = & \frac{d}{dt}\tilde{T}r_{\mathrm{SB}}\left(\frac{d{\tilde{f}}_{\mathrm{SB}}\left(\tilde{\chi },t\right)}{dt}R\right)=\tilde{T}r_{\mathrm{SB}}\left[\frac{R}{2}\left(\sum_{jk}J_{jk}\left(\nabla_{j}{\tilde{H}}_{\mathrm{SB}}\left(\tilde{\chi }\right)\right)\nabla_{k}{\tilde{f}}_{\mathrm{SB}}\left(\tilde{\chi },t\right)\right.\right.\\ & - & \left.\left.\sum_{mn}J_{mn}\left(\nabla_{m}{\tilde{f}}_{\mathrm{SB}}\left(\tilde{\chi },t\right)\right)\nabla_{n}{\tilde{H}}_{\mathrm{SB}}\left(\tilde{\chi }\right)\right)\right]={\langle \langle \frac{P}{M}\rangle \rangle }_{t}\;, \end{array}$$

where we integrated by parts and used $\partial_{R}\partial_{P}{\tilde{H}}_{\mathrm{SB}}\left(\tilde{\chi }\right)=0$.

The time derivative of ${\langle \langle P\rangle \rangle }_{t}$ is

(30) $$\begin{array}{ccc} \frac{d}{dt}{\langle \langle P\rangle \rangle }_{t} & = & \tilde{T}r_{\mathrm{SB}}\left(P\frac{d}{dt}{\tilde{f}}_{\mathrm{SB}}\left(\tilde{\chi },t\right)\right)=\tilde{T}r_{\mathrm{SB}}\left[\frac{P}{2}\left(\sum_{jk}J_{jk}\left(\nabla_{j}{\tilde{H}}_{\mathrm{SB}}\left(\tilde{\chi }\right)\right)\nabla_{k}{\tilde{f}}_{\mathrm{SB}}\left(\tilde{\chi },t\right)\right.\right.\\ & - & \left.\left.\sum_{mn}J_{mn}\left(\nabla_{m}{\tilde{H}}_{\mathrm{SB}}\left(\tilde{\chi }\right)\right)\nabla_{n}{\tilde{f}}_{\mathrm{SB}}\left(\tilde{\chi },t\right)\right)\right]=\tilde{T}r_{\mathrm{SB}}\left[\frac{P}{2}\left(\frac{\partial {\tilde{H}}_{\mathrm{SB}}\left(\tilde{\chi }\right)}{\partial R}\cdot \frac{\partial {\tilde{f}}_{\mathrm{SB}}\left(\tilde{\chi },t\right)}{\partial P}\right.\right.\\ & - & \left.\left.\frac{2P}{M}\cdot \frac{\partial {\tilde{f}}_{\mathrm{SB}}\left(\tilde{\chi },t\right)}{\partial R}+\frac{\partial {\tilde{f}}_{\mathrm{SB}}\left(\tilde{\chi },t\right)}{\partial P}\cdot \frac{\partial {\tilde{H}}_{\mathrm{SB}}\left(\tilde{\chi }\right)}{\partial R}\right)\right]\\ & = & \tilde{T}r_{\mathrm{SB}}\left(\frac{P}{M}\frac{\partial {\tilde{H}}_{\mathrm{SB}}\left(\tilde{\chi }\right)}{\partial R}\cdot \frac{\partial {\tilde{f}}_{\mathrm{SB}}\left(\tilde{\chi },t\right)}{\partial P}+\frac{P}{M}\frac{\partial {\tilde{f}}_{\mathrm{SB}}\left(\tilde{\chi },t\right)}{\partial P}\cdot \frac{\partial {\tilde{H}}_{\mathrm{SB}}\left(\tilde{\chi }\right)}{\partial R}\right)\\ & = & -{\langle \langle \frac{dV_{B}\left(R\right)}{dR}\rangle \rangle }_{t}-{\langle \langle \frac{\partial {\tilde{V}}_{\mathrm{SB}}\left(\hat{r};R\right)}{\partial R}\rangle \rangle }_{t}\;. \end{array}$$

Equations (29) and (30) give the formulation of Ehrenfest’s theorem for the averages of the phase space parameters *X*.

The average energy of the quantum subsystem is not conserved. Within the QCH is a particular type of open quantum system. If the number of phase space coordinates *N* is much greater than the number *n* of quantum particles of S (N≫n), one has an open quantum system in a classical bath. The dissipation of ${\langle \langle {\hat{H}}_{S}\rangle \rangle }_{t}$ can be calculated considering

(31) $${\langle \langle {\hat{H}}_{S}\rangle \rangle }_{t}=\tilde{T}r_{\mathrm{SB}}(\tilde{f}\left(\tilde{\chi },t\right){\tilde{H}}_{\mathrm{SB}}\left(\tilde{\chi }\right))\;,$$

from which

(32) $$\begin{array}{ccc} \frac{d}{dt}{\langle \langle {\hat{H}}_{S}\left(\hat{x}\right)\rangle \rangle }_{t} & = & -\frac{i}{\hbar }\tilde{T}r_{\mathrm{SB}}([{\tilde{H}}_{\mathrm{SB}}\left(\tilde{\chi }\right),\tilde{f}\left(\tilde{\chi },t\right)]{\hat{H}}_{S}\left(\hat{x}\right))\\ & + & \frac{1}{2}\tilde{T}r_{\mathrm{SB}}[\sum_{jk}J_{jk}(\nabla_{j}{\tilde{H}}_{\mathrm{SB}}\left(\tilde{\chi }\right))(\nabla_{k}f\left(\tilde{\chi },t\right)){\hat{H}}_{S}\left(\hat{x}\right)]\\ & - & \frac{1}{2}\tilde{T}r_{\mathrm{SB}}[\sum_{nm}J_{nm}(\nabla_{n}\tilde{f}\left(\tilde{\chi },t\right))(\nabla_{m}{\tilde{H}}_{\mathrm{SB}}\left(\tilde{\chi }\right)){\hat{H}}_{S}\left(\hat{x}\right)]\;. \end{array}$$

The last two terms on the rhs of Equation (32) are null because of the antisymmetry of ***J***. We are left with

(33) $$\begin{array}{ccc} \frac{d}{dt}{\langle \langle {\hat{H}}_{S}\left(\hat{x}\right)\rangle \rangle }_{t} & = & -\frac{i}{\hbar }\tilde{T}r_{\mathrm{SB}}([{\tilde{H}}_{\mathrm{SB}}\left(\tilde{\chi }\right),\tilde{f}\left(\tilde{\chi },t\right)]{\hat{H}}_{S}\left(\hat{x}\right))\\ & = & -\frac{i}{\hbar }\tilde{T}r_{\mathrm{SB}}({\tilde{H}}_{\mathrm{SB}}\left(\tilde{\chi }\right)\tilde{f}\left(\tilde{\chi },t\right){\hat{H}}_{S}\left(\hat{x}\right)-\tilde{f}\left(\tilde{\chi },t\right){\tilde{H}}_{\mathrm{SB}}\left(\tilde{\chi }\right){\hat{H}}_{S}\left(\hat{x}\right))\\ & = & -\frac{i}{\hbar }\tilde{T}r_{\mathrm{SB}}(\tilde{f}\left(\tilde{\chi },t\right)[{\hat{H}}_{S}\left(\hat{x}\right),{\tilde{H}}_{\mathrm{SB}}\left(\tilde{\chi }\right)])\\ & = & -\frac{i}{\hbar }\tilde{T}r_{\mathrm{SB}}\left(\tilde{f}\left(\tilde{\chi },t\right)\left[\frac{{\hat{p}}^{2}}{2m},{\tilde{V}}_{\mathrm{SB}}\left(\hat{r},R\right)\right]\right)\\ & = & -\frac{i}{\hbar }\tilde{T}r_{\mathrm{SB}}(\tilde{f}\left(\tilde{\chi },t\right)\left(\frac{\hat{p}}{2m}\cdot \left[\hat{p},{\tilde{V}}_{\mathrm{SB}}\left(\hat{r},R\right)\right]+\left[\hat{p},{\tilde{V}}_{\mathrm{SB}}\left(\hat{r},R\right)\right]\cdot \frac{\hat{p}}{2m}\right))\\ & = & -\frac{1}{2}\tilde{T}r_{\mathrm{SB}}(\tilde{f}\left(\tilde{\chi },t\right)\{\frac{\hat{p}}{m},\frac{\partial {\tilde{V}}_{\mathrm{SB}}\left(r,R\right)}{\partial r}{|}_{r\to \hat{r}}\})\\ & = & -\frac{1}{2}{\langle \langle \left\{\frac{\partial {\tilde{V}}_{\mathrm{SB}}\left(r;R\right)}{\partial r}{|}_{r\to \hat{r}}\;,\frac{p}{m}\right\}\rangle \rangle }_{t}\;. \end{array}$$

We now calculate the dissipation of the average value $\langle \langle H_{B}\rangle \rangle$ under the dynamics of the QCH.

(34) $$\begin{array}{ccc} \frac{d}{dt}{\langle \langle H_{B}\left(X\right)\rangle \rangle }_{t} & = & \tilde{T}r\left[\frac{1}{2}H_{B}\left(X\right)\left(\frac{\partial {\tilde{H}}_{\mathrm{SB}}\left(\tilde{\chi }\right)}{\partial R}\cdot \frac{\partial {\tilde{f}}_{\mathrm{SB}}\left(\tilde{\chi },t\right)}{\partial P}+\frac{\partial {\tilde{f}}_{\mathrm{SB}}\left(\tilde{\chi },t\right)}{\partial P}\cdot \frac{\partial {\tilde{H}}_{\mathrm{SB}}\left(\tilde{\chi }\right)}{\partial R}\right)\right.\\ & - & \left.H_{B}\left(X\right)\frac{P}{M}\cdot \frac{\partial {\tilde{f}}_{\mathrm{SB}}\left(\tilde{\chi },t\right)}{\partial R}\right]\\ & = & -{\langle \langle \frac{P}{M}\cdot \left(\frac{\partial {\tilde{H}}_{\mathrm{SB}}\left(\tilde{\chi }\right)}{\partial R}+\frac{\partial H_{B}\left(X\right)}{\partial R}\right)\rangle \rangle }_{t}\\ & = & -{\langle \langle \frac{P}{M}\cdot \frac{\partial {\tilde{V}}_{\mathrm{SB}}\left(\hat{r},R\right)}{\partial R}\rangle \rangle }_{t}\;. \end{array}$$

Equation (34) shows that the non-conservation of the energy of system B is due to its dissipated power because of the coupling with the quantum system S.

## 6. Concluding Remarks

In this paper, we considered the relationship between the quantum and the classical world, focusing on the existence of QCHs. Our detailed analysis supports a fundamental role of QCHs in describing phenomena concerning quantum systems. In particular, Ehrenfest’s theorem shows how classical-type variables emerge upon averaging over the positions and momenta operators of a quantum system in a time-dependent state. We derived the form of Ehrenfest’s theorem for a quantum bipartite system as a preliminary step to deduce Ehrenfest’s theorem for QCHs. It is worth remarking that the quantum–classical theory adopted is based on an operator-valued Wigner function evolving according to an antisymmetric quantum–classical bracket. Through our formalism, we found that the time derivative of the average energy of one component is given by the average of the symmetrized dissipated power not only in the case of the quantum bipartite system, but also for QCHs.

The development of efficient numerical algorithms for the stable integration of the dynamics of QCHs is a difficult problem and is the subject of ongoing research efforts also in our group. The interest in this problem arises from the fact that QCHs are very good approximations to physical systems in many situations, including the systems created by the classical measurement of quantum systems.

## Data Availability

Not applicable.

## Abbreviations

The following abbreviations are used in this manuscript:

QCH	quantum–classical hybrid
QM	quantum mechanics
OI	orthodox interpretation

## Appendix A. Ehrenfest’s Theorem in Quantum Systems

Let us consider a quantum system S with position and momentum operators $\left(\hat{r},\hat{p}\right)=\hat{x}$, respectively. We also assumed that the Hamiltonian operator is

(A1) $${\hat{H}}_{S}\left(\hat{x}\right)=\frac{{\hat{p}}^{2}}{2m}+{\hat{V}}_{S}\left(\hat{r}\right)\;.$$

The form of ${\hat{V}}_{S}\left(\hat{r}\right)$ is sufficiently general to embrace most of the situations of interest in non-relativistic quantum statistical mechanics. The density matrix of system S, ${\hat{f}}_{S}\left(\hat{x},t\right)$, obeys the quantum Liouville equation of motion:

(A2) $$\frac{d}{dt}{\hat{f}}_{S}\left(\hat{x},t\right)=-\frac{i}{\hbar }\left[{\hat{H}}_{S}\left(\hat{x}\right),{\hat{f}}_{S}\left(\hat{x},t\right)\right]\;.$$

In the Schrödinger picture, the averages of arbitrary dynamical variables O(x) are given by

(A3) $${\langle O\left(x\right)\rangle }_{t}={\mathrm{Tr}}_{S}\left({\hat{f}}_{S}\left(\hat{x},t\right)\hat{O}\left(\hat{x}\right)\right)$$

The evolution of the average value in Equation (A3) is given by

(A4) $$\begin{array}{ccc} \frac{d}{dt}{\langle O\left(x,t\right)\rangle }_{t} & = & -\frac{i}{\hbar }{\mathrm{Tr}}_{S}\left(\left[{\hat{H}}_{S}\left(\hat{x}\right),{\hat{f}}_{S}\left(\hat{x},t\right)\right]\hat{O}\left(\hat{x}\right)\right)\\ & = & -\frac{i}{\hbar }{\mathrm{Tr}}_{S}\left({\hat{H}}_{S}\left(\hat{x}\right){\hat{f}}_{S}\left(\hat{x},t\right)\hat{O}\left(\hat{x}\right)-{\hat{f}}_{S}\left(\hat{x},t\right){\hat{H}}_{S}\left(\hat{x}\right)\hat{O}\left(\hat{x}\right)\right)\\ & = & -\frac{i}{\hbar }{\mathrm{Tr}}_{S}\left({\hat{f}}_{S}\left(\hat{x},t\right)\hat{O}\left(\hat{x}\right){\hat{H}}_{S}\left(\hat{x}\right)-{\hat{f}}_{S}\left(\hat{x},t\right){\hat{H}}_{S}\left(\hat{x}\right)\hat{O}\left(\hat{x}\right)\right)\\ & = & -\frac{i}{\hbar }\mathrm{Tr}\left({\hat{f}}_{S}\left(\hat{x},t\right)[\hat{O}\left(\hat{x}\right),{\hat{H}}_{S}\left(\hat{x}\right)]\right) \end{array}$$

Considering now the two canonical operators $\hat{r}$ and $\hat{p}$, with $[\hat{r},\hat{p}]=i\hbar$, Equation (A4) gives

(A5) $$\begin{array}{ccc} \frac{d}{dt}{\langle r\rangle }_{t} & = & -\frac{i}{\hbar }\mathrm{Tr}\left({\hat{f}}_{S}\left(\hat{x},t\right)[\hat{r},{\hat{H}}_{S}\left(\hat{x}\right)]\right)\;, \end{array}$$

(A6) $$\begin{array}{ccc} \frac{d}{dt}{\langle p\rangle }_{t} & = & -\frac{i}{\hbar }\mathrm{Tr}\left({\hat{f}}_{S}\left(\hat{x},t\right)[\hat{p},{\hat{H}}_{S}\left(\hat{x}\right)]\right)\;. \end{array}$$

Using the canonical commutator of $\hat{r}$ and $\hat{p}$, together with the Dirac correspondence rule between commutators and Poisson brackets, which gives $\left[\hat{p},{\hat{V}}_{S}\left(\hat{r}\right)\right]=-i\hbar \partial {\hat{V}}_{S}\left(r\right)/\partial r$, Equations (A5) and (A6) provide the expression of the Ehrenfest theorem:

(A7) $$\begin{array}{ccc} \frac{d}{dt}{\langle r\rangle }_{t} & = & \frac{<\hat{p}{>}_{t}}{m} \end{array}$$

(A8) $$\begin{array}{ccc} \frac{d}{dt}{\langle p\rangle }_{t} & = & -{\langle \frac{d{\hat{V}}_{S}\left(r\right)}{dr}{|}_{r\to \hat{r}}\rangle }_{t} \end{array}$$

The energy of the isolated system S is clearly conserved.

## Appendix B. Operator-Valued Wigner Function, Quantum–Classical Bracket, and Partial Wigner Transform

The quantum–classical bracket can be obtained by taking the partial Wigner transform [22] over the highly massive position and momentum operator of a bipartite quantum system SB. Afterwards, an expansion of the semiclassical Moyal bracket [85] in the small adimensional parameter $m=\sqrt{m/M}<<1$ provides the quantum–classical brackets.

The quantum Liouville equation for a quantum bipartite is given in Equation (3). Upon taking the partial Wigner transform of Equation (3), one obtains

(A9) $$\begin{array}{ccc} \frac{\partial }{\partial t}{\tilde{f}}_{\mathrm{SB}}\left(X,t\right) & = & -\frac{i}{\hbar }\left({\tilde{H}}_{\mathrm{SB}}\left(X\right)e^{\frac{i\hbar }{2}\sum_{jk}{\overset{\leftarrow }{\nabla }}_{j}J_{jk}{\vec{\nabla }}_{k}}{\tilde{f}}_{\mathrm{SB}}\left(X,t\right)-{\tilde{f}}_{\mathrm{SB}}\left(X,t\right)e^{\frac{i\hbar }{2}i\sum_{mn}{\overset{\leftarrow }{\nabla }}_{m}J_{mn}{\vec{\nabla }}_{n}}{\tilde{H}}_{\mathrm{SB}(X)}\right)\;, \end{array}$$

where we used the partial Wigner transform of a product of operators:

(A10) $$\begin{array}{ccc} W_{X}{\left({\hat{O}}_{1}{\hat{O}}_{2}\right)}_{W}\left(R,P\right) & = & {\tilde{O}}_{1,\mathrm{SB}}\left(X\right)e^{\frac{i\hbar }{2}\sum_{jk}{\overset{\leftarrow }{\nabla }}_{j}J_{jk}{\vec{\nabla }}_{k}}{\tilde{O}}_{2,\mathrm{SB}}\left(R,P\right)\;, \end{array}$$

$W_{X}\left(\ldots \right)$ denotes the partial Wigner transform over $\hat{X}$. Hence, within this approach, we have ${\tilde{f}}_{SB}\left(X,t\right)=W_{X}\left({\hat{f}}_{\mathrm{SB}}\right)$ and ${\tilde{O}}_{SB}\left(X\right)={\left(2\pi \hbar \right)}^{N}W_{X}\left({\hat{O}}_{\mathrm{SB}}\right)$; see [22].

To obtain a QCH, one introduces suitable units for energy, time, length, the momentum of the light particles, and the momentum of the heavy particles:

(A11) $$\begin{array}{ccc} \Delta E & = & \Delta w_{0}\;, \end{array}$$

(A12) $$\begin{array}{ccc} \Delta \tau_{0} & = & \hbar /\Delta w_{0}\;, \end{array}$$

(A13) $$\begin{array}{ccc} \Delta \ell_{m} & = & \sqrt{\hbar^{2}/m\Delta w_{0}}\;, \end{array}$$

(A14) $$\begin{array}{ccc} \Delta p_{m} & = & m\Delta \ell_{m}/t_{0}=\sqrt{m\Delta w_{0}}\;, \end{array}$$

(A15) $$\begin{array}{ccc} \Delta P_{M} & = & \sqrt{M\Delta w_{0}}\;. \end{array}$$

The units of measure in Equations (A11)–(A14) are used to define the following dimensionless variables:

(A16) $$\begin{array}{ccc} {\hat{r}}^{\prime } & = & \hat{r}/\Delta \ell_{m}\;, \end{array}$$

(A17) $$\begin{array}{ccc} R^{\prime } & = & R/\Delta \ell_{m}\;, \end{array}$$

(A18) $$\begin{array}{ccc} {\hat{p}}^{\prime } & = & \hat{p}/\Delta p_{m}\;, \end{array}$$

(A19) $$\begin{array}{ccc} P^{\prime } & = & P/\Delta P_{M}\;, \end{array}$$

(A20) $$\begin{array}{ccc} {\hat{t}}^{\prime } & = & \hat{t}/\left(\hbar /\Delta w_{0}\right)\;, \end{array}$$

(A21) $$\begin{array}{ccc} {\hat{H}}_{W}^{\prime }\left(X^{\prime }\right) & = & \frac{{\hat{H}}_{W}\left(X\right)}{\Delta w_{0}}\;, \end{array}$$

where, of course, we defined $X^{\prime }=\left(R^{\prime },P^{\prime }\right)$. Phase space derivatives can now be defined in terms of the adimensional variables as

(A22) $$\begin{array}{ccc} \frac{\partial }{\partial t} & = & \frac{\Delta w_{0}}{\hbar }\frac{\partial }{\partial t^{\prime }} \end{array}$$

(A23) $$\begin{array}{ccc} \frac{\partial }{\partial R} & = & \frac{\sqrt{m\Delta w_{0}}}{\hbar }\frac{\partial }{\partial R^{\prime }} \end{array}$$

(A24) $$\begin{array}{ccc} \frac{\partial }{\partial P} & = & \frac{1}{\sqrt{M\Delta w_{0}}}\frac{\partial }{\partial P^{\prime }} \end{array}$$

(A25) $$\begin{array}{ccc} \frac{i\hbar }{2}\sum_{jk}{\overset{\leftarrow }{\nabla }}_{j}J_{jk}{\vec{\nabla }}_{k} & = & im\sum_{jk}{\overset{\leftarrow }{\nabla }}_{j}^{\prime }J_{jk}{\vec{\nabla }}_{k}^{\prime }\;, \end{array}$$

where $\nabla^{\prime }=\left(\left(\partial /\partial R^{\prime }\right),\left(\partial /\partial P^{\prime }\right)\right)$. Then, Equation (A9) becomes

(A26) $$\begin{array}{ccc} \frac{\partial }{\partial t^{\prime }}{\tilde{f}}_{\mathrm{SB}}^{\prime }\left(X^{\prime },t^{\prime }\right) & = & -i\left({\tilde{H}}_{\mathrm{SB}}^{\prime }\left(X^{\prime }\right)e^{\frac{im}{2}\sum_{jk}{\overset{\leftarrow }{\nabla }}_{j}^{\prime }J_{jk}{\vec{\nabla }}^{\prime }}{\tilde{f}}_{\mathrm{SB}}^{\prime }\left(X^{\prime },t^{\prime }\right)\right.\\ & - & \left.{\tilde{f}}_{\mathrm{SB}}\left(X^{\prime },t^{\prime }\right)e^{\frac{im}{2}\sum_{mn}{\overset{\leftarrow }{\nabla }}_{m}^{\prime }J_{mn}{\vec{\nabla }}_{n}^{\prime }}{\tilde{H}}_{\mathrm{SB}}\left(X^{\prime }\right)\right)\;. \end{array}$$

Since m<<1, we can expand the exponential up to the first order:

(A27) $$e^{\frac{im}{2}\sum_{jk}{\overset{\leftarrow }{\nabla }}_{j}^{\prime }J_{jk}{\vec{\nabla }}_{k}^{\prime }}\approx 1+\frac{im}{2}\sum_{jk}{\overset{\leftarrow }{\nabla }}_{j}^{\prime }J_{jk}{\vec{\nabla }}_{k}^{\prime }+\ldots$$

Hence, we obtain the quantum–classical equation of motion in scaled coordinates:

(A28) $$\begin{array}{ccc} \frac{\partial }{\partial t^{\prime }}{\tilde{f}}_{\mathrm{SB}}^{\prime }\left(X^{\prime },t^{\prime }\right) & = & -i[{\tilde{H}}_{\mathrm{SB}}^{\prime },{\tilde{f}}_{\mathrm{SB}}^{\prime }\left(X^{\prime },t^{\prime }\right)]+\frac{m}{2}\left(\sum_{jk}{\tilde{H}}_{\mathrm{SB}}^{\prime }\left(X^{\prime }\right){\overset{\leftarrow }{\nabla }}_{j}^{\prime }J_{jk}{\vec{\nabla }}_{k}^{\prime }{\tilde{f}}_{\mathrm{SB}}^{\prime }\left(X^{\prime },t^{\prime }\right)\right)\\ & - & \frac{m}{2}\left(\sum_{mn}{\tilde{f}}_{\mathrm{SB}}^{\prime }\left(X^{\prime },t^{\prime }\right){\overset{\leftarrow }{\nabla }}_{m}^{\prime }J_{mn}{\vec{\nabla }}_{n}^{\prime }{\tilde{H}}_{\mathrm{SB}}^{\prime }\left(X^{\prime }\right)\right)\;. \end{array}$$

Going back to dimensional coordinates, one finally obtains Equation (11).
